# RECRUITING LATINO CAREGIVERS INTO DEMENTIA-RELATED RESEARCH: NOT FOR THE FAINT OF HEART

**DOI:** 10.1093/geroni/igae098.1409

**Published:** 2024-12-31

**Authors:** Maria Aranda

**Affiliations:** University of Southern California, Los Angeles, California, United States

## Abstract

Drawing from the theoretical model—the Micro-Meso-Macro Framework— we elucidate factors that facilitated or impeded diverse sample recruitment to a community-based caregiver intervention study with high Hispanic/Latino representation in California. Based on our multi-level analysis of recruitment meetings and community outreach tracking, we documented the following factors implicated in trial participation: 1) Micro: prior encounters with the research enterprise; educational attainment and occupational status; sense of altruism and beneficence; monetary incentivization; comfort with disclosure of private matters; starting from ground zero; gender issues; and 2) Meso/macro: assembling a proactive scientific team; incorporating a marketing perspective; leveraging logos and reputation; training trial staff and community partners; writing project budgets that incentivize diverse trial recruitment; making inroads with community partners; navigating technological challenges; and tapping into resources to alleviate barriers. Latinos were adversely affected by low exposure to research activities and technological barriers which necessitated extra supports during trial participation.

